# Structures of Arenaviral Nucleoproteins with Triphosphate dsRNA Reveal a Unique Mechanism of Immune Suppression*[Fn FN2]

**DOI:** 10.1074/jbc.M112.420521

**Published:** 2013-04-24

**Authors:** Xue Jiang, Qinfeng Huang, Wenjian Wang, Haohao Dong, Hinh Ly, Yuying Liang, Changjiang Dong

**Affiliations:** From the ‡ Norwich Medical School, University of East Anglia, Norwich Research Park, Norwich NR4 7TJ, United Kingdom,; the § Department of Veterinary and Biomedical Sciences, University of Minnesota, Twin Cities, St. Paul, Minnesota 55108,; the ¶ Laboratory of Department of Surgery, the First Affiliated Hospital, Sun Yat-sen University, 58 Zhongshan Road II, Guangzhou, Guangdong 510080, China, and; the ‖ Biomedical Sciences Research Complex, University of St. Andrews, St. Andrews KY16 9ST, United Kingdom

**Keywords:** Immunosuppression, Nucleic Acid Enzymology, Protein Structure, Protein-nucleic Acid Interaction, Viral Protein, 3′-5′ Exoribonuclease, Lassa Fever Virus, Tacaribe Virus, Type I Interferons, Immune Evasion

## Abstract

A hallmark of severe Lassa fever is the generalized immune suppression, the mechanism of which is poorly understood. Lassa virus (LASV) nucleoprotein (NP) is the only known 3′-5′ exoribonuclease that can suppress type I interferon (IFN) production possibly by degrading immune-stimulatory RNAs. How this unique enzymatic activity of LASV NP recognizes and processes RNA substrates is unknown. We provide an atomic view of a catalytically active exoribonuclease domain of LASV NP (LASV NP-C) in the process of degrading a 5′ triphosphate double-stranded (ds) RNA substrate, a typical pathogen-associated molecular pattern molecule, to induce type I IFN production. Additionally, we provide for the first time a high-resolution crystal structure of an active exoribonuclease domain of Tacaribe arenavirus (TCRV) NP. Coupled with the *in vitro* enzymatic and cell-based interferon suppression assays, these structural analyses strongly support a unified model of an exoribonuclease-dependent IFN suppression mechanism shared by all known arenaviruses. New knowledge learned from these studies should aid the development of therapeutics against pathogenic arenaviruses that can infect hundreds of thousands of individuals and kill thousands annually.

## Teaser

**Background:** Arenaviral nucleoproteins play a critical role in innate immune suppression.

**Results:** Structures of Lassa nucleoprotein in complex with triphosphate dsRNA and Tacaribe virus nucleoprotein have been determined.

**Conclusion:** Both Lassa and Tacaribe nucleoproteins can strongly inhibit IFN-β production by degrading immune-stimulatory dsRNA.

**Significance:** A unique immune suppression mode of arenaviral nucleoproteins has been revealed.

## Introduction

Arenaviruses naturally infect animals (mostly rodents) and can cause severe hemorrhagic fever infections in humans with limited preventative and therapeutic measures (1). Lassa fever virus (LASV),^5^ for example, infects ∼500,000 people and causes ∼5,000 deaths annually in endemic areas of West Africa (2). Several South America arenaviruses cause hemorrhagic fever outbreaks with a mortality rate up to 30% (1, 3). Due to increased human contact with animals and frequent international travel, pathogenic arenaviruses pose emerging threats to public health and national security.

A hallmark of severe arenavirus-induced hemorrhagic fevers is the general immune suppression of hosts that leads to uncontrolled viral replication in various organs (4, 5). Although the exact mechanisms by which these viruses induce generalized immune suppression are not well understood, several studies have shown that most arenavirus NP proteins, except for that of the Tacaribe virus (TCRV), can strongly suppress the induction of type I interferons (IFNs) (6, 7). We have provided strong evidence in the current study to show that the TCRV NP protein can also inhibit IFNs, which is contradictory to previous reports.

NP is a multifunctional protein involved in viral genomic RNA encapsidation, viral RNA synthesis, and immune evasion. We reported the first crystal structure of a full-length LASV NP protein (8), which has uncovered a unique molecular mechanism of NP in mediating IFN suppression. Our structural, biochemical, and cell-based analyses have demonstrated that NP encodes a functional 3′-5′ exoribonuclease of the DEDDH family at its C terminus and that this RNase activity plays a strikingly important role in IFN suppression (8). Our findings have subsequently been corroborated by an independent study on the structural and biochemical characterization of the C-terminal domain of LASV NP (9). We proposed a novel immune evasion mechanism by which LASV NP preferentially degrades the immune stimulatory RNAs to avoid the induction of type I IFNs (8). To our knowledge, no other known host or viral RNase has a similar function in suppressing type I IFN induction. The closest equivalent would be the cellular DNA exonuclease Trex1, which inhibits innate immunity by degrading ssDNA and dsDNA templates that would otherwise be recognized by the cellular DNA sensors to induce IFN production (10, 11). How this unique exoribonuclease activity of LASV NP recognizes and degrades the immune-stimulatory RNAs to avoid IFN induction remains a mystery.

While this manuscript was in preparation, a 2.9-Å structure of a catalytically inactive mutant version of LASV NP-C in complex with a dsRNA was reported (12). At this resolution, however, it was not possible to visualize the sequence of the dsRNA and water molecules in the active site as well as to identify all possible side chain interactions. Additionally, using an inactive mutant enzyme limits the ability to understand how this unique exoribonuclease cleaves dsRNA. In the current study, we provide an atomic view of the intermediate conformation of the active 3′-5′ exoribonuclease domain of LASV NP in the process of degrading a 5′-triphosphate dsRNA template, which is a typical pathogen-associated molecular pattern molecule. Together with the time course soaking crystallographic study, our structural analyses provide important and novel insights into the mechanism of this unique exoribonuclease in binding and cleaving its substrates. Furthermore, we have provided compelling structural, enzymatic, and biological data to demonstrate that, in contrast to a long-standing belief, the TCRV NP protein closely resembles LASV NP in the tertiary structure, exoribonuclease enzymatic activity, and the IFN suppressive function. Collectively, these data support a novel and unified model for arenaviruses to suppress IFN production via the unique exoribonuclease activity of viral NP protein.

## EXPERIMENTAL PROCEDURES

### 

#### 

##### Plasmids and Cells

For bacterial cell expression of the LASV NP C-terminal domain (LASV NP-C), the fragment spanning residues 364 to 569 of LASV NP was subcloned into the pLou3 plasmid. The full-length TCRV NP gene was kindly provided by J. Nunberg, University of Montana. For bacterial expression of the TCRV NP-C domain, the fragment of residues 364 to 569 was cloned into the pHisTEV plasmid. For protein expression in mammalian cells, full-length LASV or the TCRV NP gene was cloned into the pCAGGS expression vector. 293T cells were grown in high-glucose DMEM supplemented with 10% fetal bovine serum (FBS).

##### Protein Expression and Purification

Full-length LASV NP and NP mutants were expressed in *Escherichia coli* from pLou3-based plasmids and purified as previously described (8). LASV NP-C protein was expressed in Rosetta cells after induction with 0.1 mm isopropyl 1-thio-β-d-galactopyranoside overnight at 20 °C and purified as previously described (8). TCRV NP-C protein expression was induced overnight by 0.3 mm isopropyl 1-thio-β-d-galactopyranoside at 20 °C and purified through a nickel column followed by a gel filtration column.

##### RNA Oligos

RNA oligos used in this study were either chemically synthesized (5′-hydroxylate, 5′-OH) or *in vitro* transcribed (5′-triphosphate, 5′-ppp) using the MEGAscript kit (Ambion). The dsRNA substrate used in the co-crystallization was generated by hybridization of an 8-nt 5′-triphosphate RNA oligo 5′-pppGGGCGCCCC-3′ by itself in a hybridization buffer that contains 20 mm Tris, pH 8.0, 0.1 m NaCl, and 1 mm EDTA. The 15-nt RNA oligos used in the *in vitro* RNase assay, 5′-AGUAGAAACAAGGCC-3′, were chemically synthesized, and 5′ labeled with [γ-^32^P]ATP using a T4 polynucleotide kinase (New England Biolabs).

##### Crystallization and Data Collection

The LASV NP-C protein was mixed with the 5′-triphosphate dsRNA substrate at a 1:1 molar ratio on ice for 30 min. Although several dsRNAs of different sizes could form crystals with LASV NP-C, the best crystals were obtained with the 8-bp 5′-triphosphate dsRNA. These crystals were obtained in 18.5% PEG MME 5000, 0.1 m sodium citrate, pH 4.5, and 2.4% PEG MME350. Crystals of TCRV NP-C were grown in 32% PEG3350, 0.1 m MOPS, pH 7.0, and 0.11 m ammonium citrate. The data were collected at beamlines IO2, IO3, and IO4 of Diamond light resources, processed using MOSFLM (13), merged, and scaled using SCALA in the CCP4 suite (14). The data collection statistics are listed in Table 1.

##### Structural Determination and Refinement

All structures were determined by molecular replacement using Phaser (14, 15) and a search model of the LASV NP C-terminal domain (PDB code 3MWP). The models were built using Coot (16) and structural refinements were carried out using REFMAC5 (17) with detwinning at the last cycle. The structures were evaluated using Molproperty and structural statistics are listed in Table 1.

##### Soaking Crystallography

The crystals of NP-C coupled with dsRNA were soaked at room temperature in cryoprotectant containing 20% glycerol, 18.5% PEG MME 5000, 0.1 m sodium citrate, pH 5.5, 2.4% PEG MME350, and 100 mm MnCl_2_ for 1, 1.5, 5, and 10 min, respectively. The crystals were frozen in liquid nitrogen for data collection. The data collection, integration, and structural determination were performed as described above.

##### Site-directed Mutagenesis

Single, double, and triple point mutations were generated using either the QuikChange protocol (Stratagene) or overlapped PCR. All the mutations have been confirmed by DNA sequencing.

##### In Vitro 3′-5′ Exoribonuclease Assay

The 15-nt RNA oligo was 5′ labeled with ^32^P in a polynucleotide kinase reaction and unincorporated [γ-^32^P]ATP was removed by passing through the Illustra MicroSpin G25 column (GE Healthcare Life Sciences). The 5′ ^32^P-labeled RNA oligo was annealed with a 5-fold amount of unlabeled RNA oligos of either the same or the complementary oligo to form either ssRNA or dsRNA substrates. The exoribonuclease reaction contained 1 pmol of radiolabeled RNA substrates in either ss or ds forms and various concentrations of NP proteins (1 to 100 nm) in an RNase buffer (25 mm Tris-HCl, pH 7.5, 10 mm NaCl, 15 mm KCl, 1 mm MnCl_2_, and 0.1 mg/ml of bovine serum albumin) and incubated at 37 °C for 60 min. EDTA was added to a final concentration of 10 mm to terminate the reaction. Radiolabeled RNA products were heated at 95 °C for 5 min, rapidly cooled on ice, separated in a 17% urea-polyacrylamide gel, and visualized by exposure onto an x-ray film.

##### Sendai Virus-induced IFN Production Assay

Using a calcium phosphate transfection method, 293T cells were co-transfected with 100 ng of a plasmid that expresses the firefly luciferase (FLuc) gene from the IFN-β promoter (pIFNb-luc), different amounts (10 and 100 ng) of LASV NP or TCRV NP expression vectors, as well as 50 ng of the β-galactosidase (β-gal) expressing plasmid for normalization purpose. At 24 h post-transfection, cells were infected with Sendai virus at a multiplicity of infection of 1 to induce IFN expression. At 24 h post-infection, cell lysates were prepared for luciferase and β-Gal assays (Promega). FLuc levels were normalized by β-Gal values.

##### Statistical Analyses

Statistical analyses were conducted using the Student's *t* test.

## RESULTS

### 

#### 

##### LASV NP 3′-5′ Exoribonuclease Preferentially Degrades dsRNA

**FIGURE 1.**
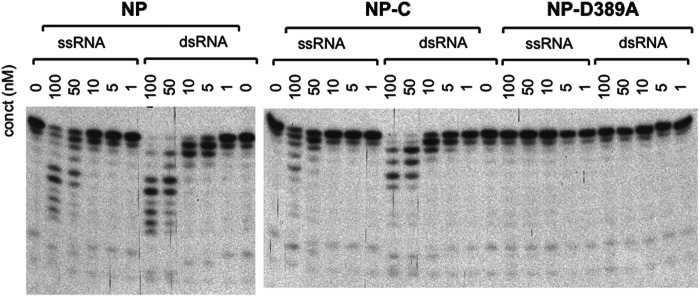
**LASV NP exoribonuclease activity preferentially degrades dsRNA substrates.** Various concentrations of purified full-length NP, C-terminal domain of NP (*NP-C*), and a catalytic mutant (*NP-D389A*) were incubated at 37 °C for 60 min with a 5′ ^32^P-labeled 15-nt RNA oligo of either ss or ds forms (“Experimental Procedures”). The products were separated in a 17% urea-polyacrylamide gel and exposed to films. A representative gel of at least three independent experiments was shown.

To identify the preferred RNA substrates of LASV NP exoribonuclease, we first compared the efficiency of LASV NP in degrading ssRNA *versus* dsRNA substrates. Synthetic 15-nt RNA oligos were radiolabeled with ^32^P at the 5′ end, and incubated with unlabeled RNA oligos of either the same or complementary strand to form either ssRNA or dsRNA substrates. The *in vitro* exoribonuclease assay was conducted at a predetermined optimal condition (8) with various concentrations of full-length LASV NP, NP C-terminal domain (NP-C) of residues 364 to 569, and catalytic mutant D389A. NP and NP-C degraded dsRNA substrate at a much lower concentration than ssRNA (by at least 10-fold) (Fig. 1*A*). As a control, the NP-D389A mutant, in which the first aspartate of the catalytic residues (DEDDH) was changed to alanine, completely lost the ability to cleave any of the RNA species, which is consistent with our previous observation (8). The integrity of the NP proteins during the *in vitro* RNase assay was demonstrated by SDS-PAGE analysis of the proteins after 0 and 90 min incubation at 37 °C (supplemental Fig. S1). Our data clearly demonstrate that dsRNA is the preferred substrate for NP RNase activity and that the NP-C domain alone has similar substrate selectivity and catalysis as the full-length protein. This dsRNA substrate preference of LASV NP exoribonuclease activity correlates well with the fact that dsRNA is a typical pathogen-associated molecular pattern molecule to induce type I IFN production (18, 19).

##### Structure of Active LASV NP-C RNase in Complex with 5′ Triphosphate dsRNA

**FIGURE 2.**
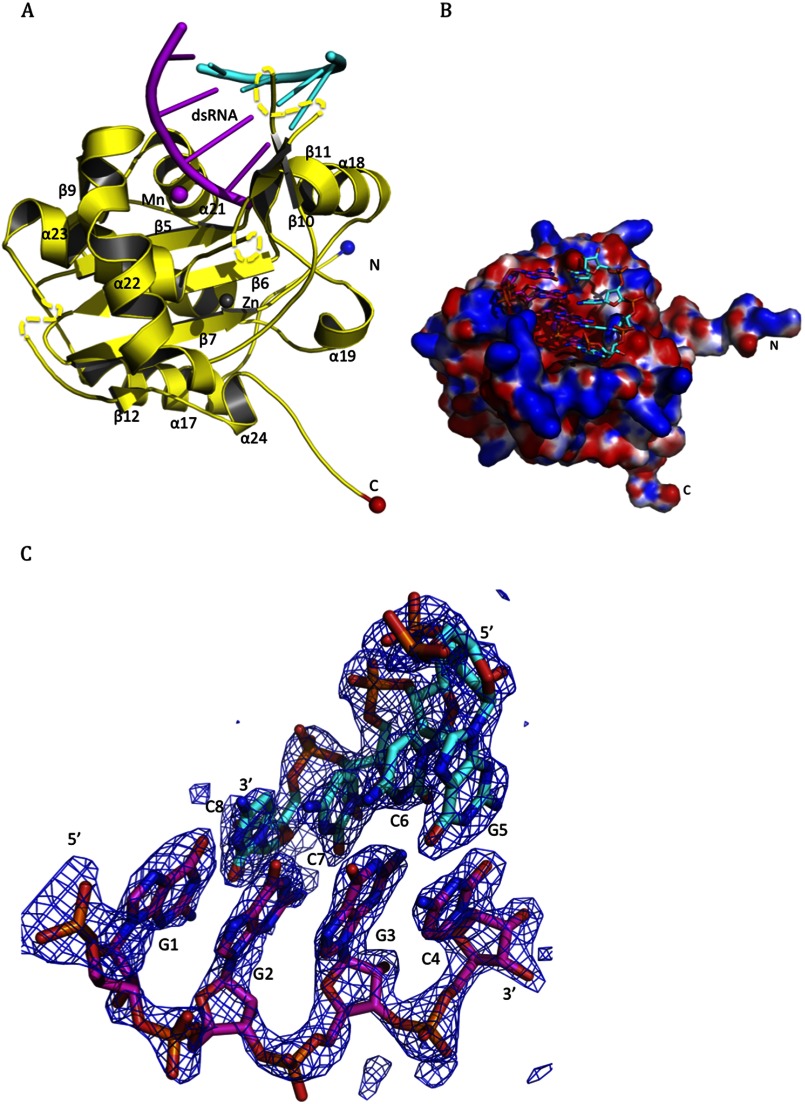
**Crystal structure of the active RNase domain of LASV NP in complex with the 5′-ppp dsRNA oligo.** *A,* in the LASV NP-C·dsRNA complex structure, LASV NP-C is shown in *yellow* for the backbone, with a *blue ball* indicating the N terminus and a *red ball* indicating the C terminus, whereas the dsRNA substrate is shown in *magenta* for the cleaving strand and in *cyan* for the complementary strand. Positions of the α-helices and β-sheets are indicated. A Mn^2+^ ion is shown as a *magenta ball* and a zinc ion as a *gray ball. B,* electrostatic surface potential map of the LASV NP-C domain in complex with the dsRNA substrate (shown in a stick model). *Red* and *blue* indicate negatively and positively charged surface potentials, respectively. *C,* the *F_o_* − *F_c_* electron density map contoured at 2.5 σ is shown in *blue mesh* for the dsRNA substrate. The dsRNA is shown in *stick* with the cleaving strand in *magenta* and the complementary strand in *cyan*.

To understand the molecular basis for the NP RNase substrate selectivity and enzymatic mechanism, we attempted to co-crystallize the catalytically active LASV NP-C domain with different dsRNA substrates. We obtained crystals of NP-C in the presence of an 8-bp dsRNA oligo (5′-GGGCGCCC-3′/3′-CCCGCGGG-5′) with either 5′-OH or 5′-triphosphate (5′-ppp), with the 5′-ppp dsRNA complex diffracted to a much higher resolution (1.68 Å). These crystals belong to a space group of P4_1_2_1_2 with unit cell dimensions of *a* = 46.74 Å, *b* = 46.74 Å, *c* = 217.31 Å, and α = β = γ = 90°. The complex structure was determined by molecular replacement using Phaser (14, 15) with an initial search model of the C-terminal domain of the full-length NP (PDB code 3MWP), and refined to *R*_factor_ of 0.20 and *R*_free_ of 0.22. Single asymmetric unit cells contain one NP-C monomer bound to an RNA substrate (Fig. 2*A*). The electrostatic surface potential map shows that the dsRNA-binding surface, with a surface area of 606 Å^2^, is highly positively charged (Fig. 2*B*).

Superimposition of Cα atoms of NP-C with and without RNA substrate (*i.e.* the current structure and one in the PDB 3MWP, respectively) showed highly similar overall structures, with a root mean square deviation of 0.8554 Å from residues 364 to 561 (supplemental Fig. 2). Nevertheless, in the presence of RNA substrate, the side chains of a few residues (*e.g.* Tyr-429, Arg-393, Asp-465, Lys-488, Arg-492, Ser-430, Gln-462, and Lys-469) showed significant conformational changes, suggesting their potential roles in substrate binding and processing. The five catalytic residues (Asp-389, Glu-391, His-522, Asp-466, and Asp-533) also showed some minor conformational changes. In addition, compared with the RNA-free NP structure (PDB 3MWP), residues 562 to 566 at the C-terminal end in the NP-C·dsRNA complex folded into an ordered conformation (supplemental Fig. S2).

**FIGURE 3.**
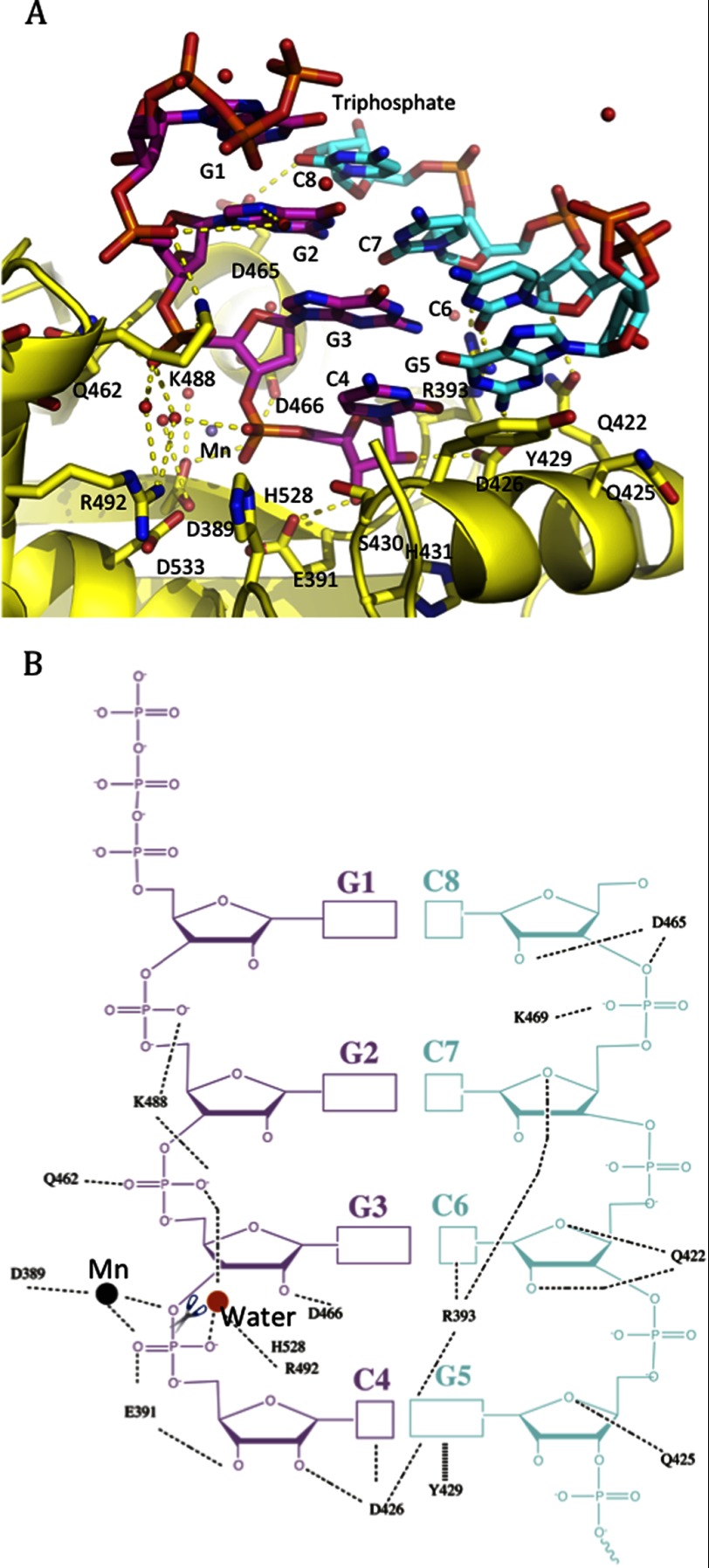
**The RNA-binding interface within the LASV NP RNase domain.** *A*, the dsRNA substrate bound in the surface cleft is shown as a *stick* model. The cleaving strand of dsRNA is shown in *magenta* and the complementary strand in *cyan. B,* schematic representation of the 4-bp dsRNA substrate and its interacting residues within the LASV NP RNase domain. The scissile bond is indicated with a *scissor*. Mn^2+^ is shown as a *black ball*.

Electron density of the RNA substrate clearly showed the presence of 4 bp (5′-GGGC-3′/3′-CCCG-5′) in the catalytic cavity (Fig. 2*C*). Similarly, the 3′-5′ exonucleases Trex1 and Trex2 also contain 4 bases of their ssDNA substrates in the catalytic pockets (20–22). The dsRNA substrate in the LASV NP-C catalytic site corresponds to the first 4 bp (5′-GGGC-3′/3′-CCCG-5′) of the input 8-bp dsRNA (5′-GGGCGCCC-3′/3′-CCCGCGGG-5′. For convenience, the 8-bp RNA is numbered as 5′-G1G2G3C4G5C6C7C8–3′ (Fig. 3*A*). This was because a catalytically active NP-C domain was used in the co-crystallization process and was predicted to have removed the last 4 nucleotides from the 3′ end of the cleaving strand, resulting in an intermediate dsRNA product with a 5′ protruding end of the complementary strand (5′-GGGC-3′/3′-CCCGCGGG-5′). The 4 nucleotides 5′-GGGC-3′ of the cleaving strand were almost completely buried in the catalytic cavity (Fig. 3*A*). Residues Asp-426, Glu-391, Asp-466, Gln-462, and Lys-488 in the cavity interacted directly with the cleaving stand, whereas other residues in the cavity coordinated the binding indirectly through water molecules (Fig. 3, *A* and *B*). Most of the residues interacting with the cleaving strand are phylogenetically conserved among arenaviruses, such as Gly-392, His-431, Glu-391, Asp-466, Gln-462, Arg-492, and Ser-430. In particular, the 3′-end ribonucleotide C at position 4 of the cleaving strand was coordinately anchored by several residues: Arg-492 through a water molecule forming a hydrogen bridge with the phosphate group, and Arg-426 and Glu-391 through their side chains forming a hydrogen bond with the 2′- and 3′-oxygen of the ribose of the cytosine ribonucleoside, respectively (Fig. 3*B*). Gln-462 and Asp-466 bound the guanine ribonucleotide (G) at the 3 position through the side chains forming a hydrogen bond with the phosphoryl oxygen and 2′-oxygen of the ribose (Fig. 3*B*). Lys-488 formed two salt bridges with the phosphates of the two Gs at the 3 and 2 positions, respectively (Fig. 3*B*). Meanwhile, the 3′-CCCG-5′ nucleotides of the complementary strand were stacked against the aromatic side chain of Tyr-429 and were anchored by the side chains of residues Arg-393, Gln-425, Asp-426, Gln-422, and Asp-465, whereas the rest of the nucleotides of the complementary strand (3′-CGGG-5′) were exposed to solvent (Fig. 3*B*). Interestingly, residues involved in the complementary strand binding are not phylogenetically conserved.

Remarkably, the 3′-end ribonucleotide C of the cleaving strand was positioned precisely in the deep pocket of the catalytic site, with the terminal bond of the ribonucleotide completely exposed to the catalytic residues for hydrolysis. This provides strong structural evidence for LASV NP being a 3′-5′ exoribonuclease as has recently been proposed (8, 9). Unlike some other known 3′-5′ exonucleases, such as Trex1, Trex2, and Klenow fragment (20, 22, 23), in which the single-stranded DNA was present in the catalytic pocket (even though a hairpin DNA was used in co-crystallization with Klenow), the NP-C·dsRNA complex structure contained dsRNA in the active site with the 3′-end ribonucleotide C remaining double-stranded and presented directly to the catalytic residues (Fig. 3), suggesting that the NP exoribonuclease activity does not need to separate the two annealing strands prior to cleaving the 3′-end ribonucleotide.

##### Effects of Mutating Key Residues of LASV NP Important for dsRNA Substrate Binding and Cleavage in Modulating RNase Activity and IFN Suppression

**FIGURE 4.**
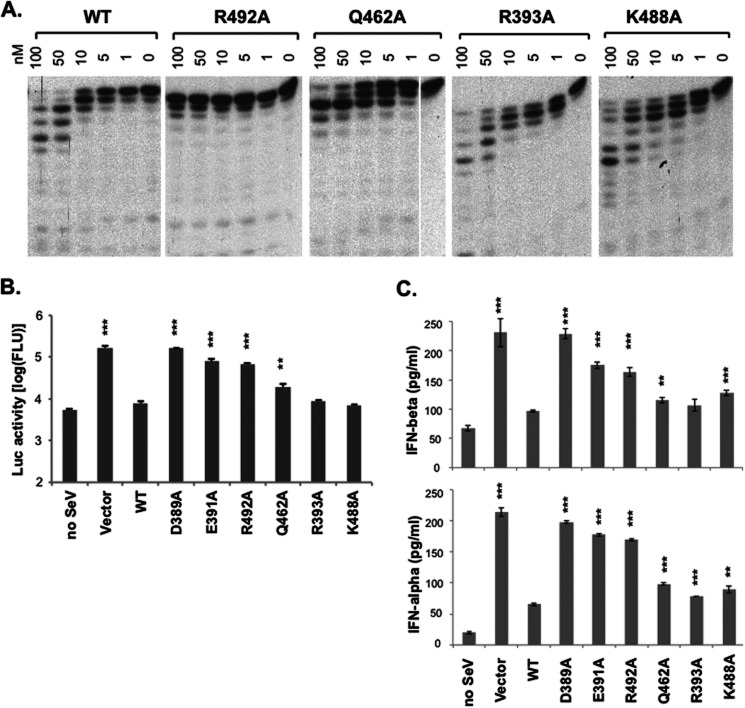
**Mutational analyses of the residues identified for dsRNA binding and processing by the *in vitro* RNase assay and cell culture-based IFN suppression assay.** *A,* WT and mutant LASV NP proteins were assessed for their exoribonuclease function in the *in vitro* RNase assay. A representative gel of at least three independent experiments was shown for each of the proteins. *B,* the ability of WT and mutant LASV NP proteins to suppress Sendai virus-induced IFN production was determined by a LUC-based IFN-β promoter assay. The results shown are the average of three independent experiments with *error bars* showing S.D. *C,* the levels of IFN-β and IFN-α produced from Sendai virus-infected A549 cells, in the presence of WT or mutant LASV NP proteins, were quantified by ELISA. The results shown are the average of three independent experiments with *error bars* showing S.D. Statistical analyses between the WT and mutant NP proteins were conducted using Student's *t* test with: ***, *p* < 0.001; **, *p* < 0.01.

To confirm our structural observations, we generated LASV NP-C proteins with the following individual substitutions: R492A, Q462A, R393A, or K488A. Purified mutant NP proteins were analyzed by the *in vitro* exoribonuclease assay with dsRNA substrate as described above. The integrity of the NP proteins during the *in vitro* RNase assay was demonstrated in supplemental Fig. S1. WT NP at concentrations of 50 and 100 nm resulted in RNA products that were much shorter than the 15-bp input substrate (Fig. 4*A*). In contrast, R492A failed to trim the RNA substrate even at the highest protein concentration used (100 nm), suggesting that it completely lacked RNase activity (Fig. 4*A*). Although Q462A could efficiently trim the last two ribonucleotides from the 3′ end of the 15-bp input RNA substrates, it generated a major product of 13-nt length even at the highest protein concentration used (100 nm), suggestive of an aberrant RNase property (Fig. 4*A*). However, single alanine substitution at residues Arg-393 and Lys-488, which are non-conserved among arenaviruses, did not show any appreciable defects in RNase activity (Fig. 4*A*). In summary, mutational analyses of LASV NP in the *in vitro* RNase assay have demonstrated that the phylogenetically conserved residues involved in dsRNA substrate binding played important roles in the exoribonuclease activity of NP.

As the RNase activity of LASV NP is required for its IFN suppressive function, we asked whether these mutants also affect the ability of NP to suppress IFN induction. In Sendai virus-induced activation of the IFN-β promoter assay (Fig. 4*B*), WT NP could efficiently block IFN-β induction, whereas catalytic mutants D389A and E391A, as well as the R492A mutant that completely lacked RNase activity *in vitro*, failed to suppress IFN induction. Mutant Q462A, with defective RNase activity *in vitro*, also produced significantly higher IFN than the WT protein (*p* < 0.01). On the other hand, single mutants R393A and K488A, with no obvious effect on RNase activity *in vitro*, could strongly suppress IFN production with no significant difference from the WT. In addition, we also used ELISA to directly quantify the levels of IFN-α and IFN-β production from Sendai virus-infected human lung epithelial A549 cells, in the presence of either WT or mutant NP proteins (Fig. 4*C*). As expected, WT NP effectively decreased the levels of virus-induced IFN production by more than 50%, whereas the RNase-deficient NP mutants, D389A, E391A, and R492A, produced much higher levels of IFNs that were almost comparable with that of the vector control. In contrast, Q462A, R393A, and K488A, which showed little or no reduction in the *in vitro* RNase enzymatic activities, also reduced IFNs production by nearly 50%, although it was still statistically higher than that of the WT (except for IFN-β production of the R393A mutant). In summary, we show that the activity of the NP RNase generally correlates with its ability to suppress the induction of type I IFNs.

##### TCRV NP Resembles LASV NP in Structure, Enzymatic Activity, and IFN Suppressive Function

**FIGURE 5.**
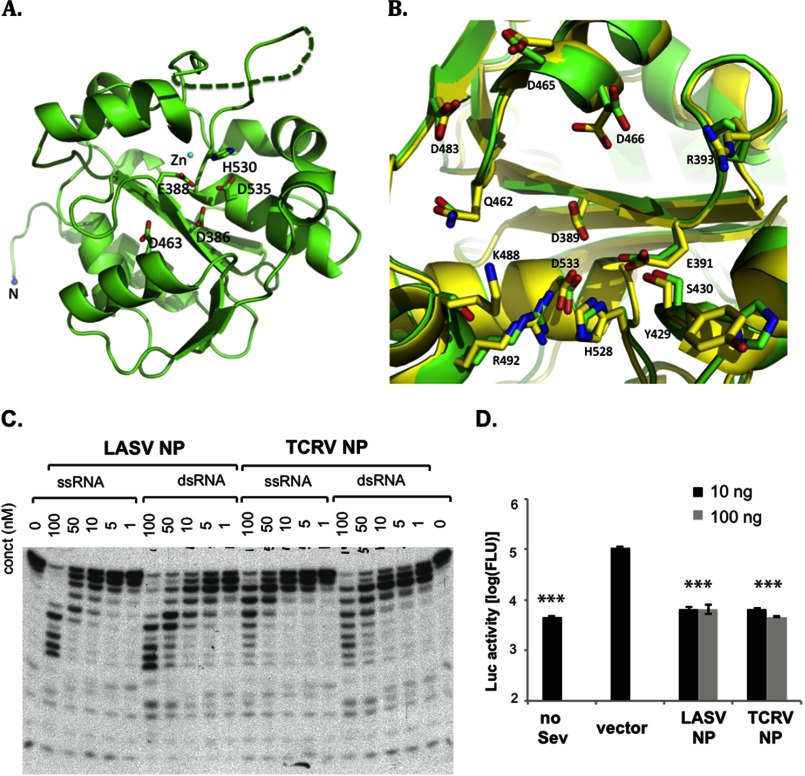
**TCRV NP resembles LASV NP in the tertiary structure, exoribonuclease activity, and IFN suppressive function.** *A,* schematic diagram of TCRV NP-C structure, with the five catalytic residues shown in a *stick* model. *B,* superimposition of LASV and TCRV NP-C structures, shown in *yellow* and *green*, respectively. The residue numbers are based on LAVS nucleoprotein. The Arg-393, Tyr-429, and Lys-488 in LASV NP are replaced by Pro-390, His-426, and Ser-485 in TCRV NP, respectively. *C*, TCRV NP has similar 3′-5′ exoribonuclease activity with dsRNA substrate preference as LASV NP. A representative gel of at least three independent experiments was shown for each protein. *D,* TCRV and LASV NPs can equally suppress Sendai virus-induced IFN production in the LUC-based promoter assay. The results shown are the average of three independent experiments with *error bars* showing S.D. Statistical analyses were conducted using the Student's *t* test. ***, *p* < 0.001.

It has previously been reported that TCRV NP differs from other arenaviral NPs in the ability to suppress IFN induction (7) despite having the same five catalytic residues in the RNase pocket. We and other researchers have noted that there is an error in the GenBank^TM^ sequence of the TCRV NP gene that results in alterations at positions 389–393. The correct sequence of TCRV NP, as verified by us and other investigators (24)^6^ is very similar to other arenaviral NPs. To determine the structural basis for the possible differential activity of TCRV NP in IFN suppression, we crystallized the TCRV NP-C domain (residues 364–569) and determined its three-dimensional structure at 1.8 Å (Fig. 5*A*). The overall structure of TCRV NP-C was very similar to that of LASV NP-C with a root mean square deviation of 1.30 Å from residue 364 to 561 (supplemental Fig. S3). The five conserved catalytic residues from TCRV NP-C were located at almost identical positions as those of the LASV NP-C with some minor conformational changes (Fig. 5*B*). The potential dsRNA-interacting interface of TCRV NP also largely resembled that of LASV NP. Specifically, residues Arg-489, Gln-459, and Ser-427 in TCRV NP were located at identical positions as the corresponding residues Arg-492, Gln-462, and Ser-430 of the LASV NP, which coordinated the binding of the cleaving strand. A noted difference was that residues Ser-485, Gly-423, and Pro-390 in TCRV NP were located at the corresponding positions of residues Lys-488, Asp-426, and Arg-393 of the LASV NP, respectively (Fig. 5*B*). It is noteworthy that these residues are not phylogenetically conserved, and that single substitution at these residues in LASV NP did not significantly affect the exoribonuclease activity of the proteins *in vitro* or their IFN suppression in cells (Fig. 4).

We next compared the RNase enzymatic and IFN suppressive functions between TCRV NP and LASV NP. We found that TCRV NP could process both ss- and dsRNA templates with similar efficiency and substrate preference as those of the LASV NP (Fig. 5*C*). Given the important role of the activity of NP RNase in IFN suppression, we decided to revisit the role of TCRV NP in IFN suppression using the same luciferase (LUC)-based assay as described previously (7, 8). Both TCRV and LASV NPs, at various concentrations, could suppress Sendai virus-induced IFN-β production at similar levels (Fig. 5*D*). Taken together, our data strongly suggest that TCRV NP, like the NP proteins of LASV and other arenaviruses (7), has active 3′-5′ exoribonuclease activity and can effectively suppress IFN production, results that are in sharp contrast to the previously published report (7). Therefore, we propose that the NP exoribonuclease-based IFN suppression represents a common mechanism for all known arenaviruses.

##### Time Course Experiment Reveals a Unique and Novel Enzymatic Mechanism of the 3′-5′ Exoribonucleolytic Activity of NP

**FIGURE 6.**
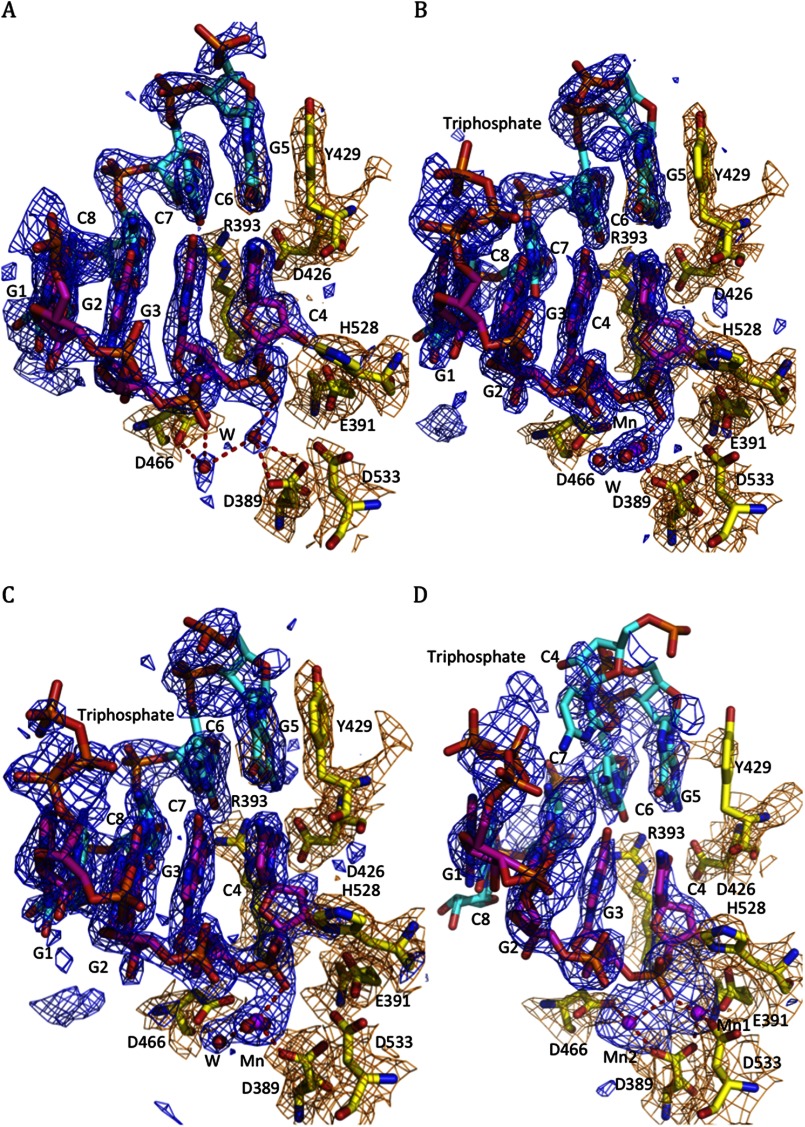
**Time course structural analysis of LASV NP-C·dsRNA complex soaked in Mn^2+^ solution.** *F_o_* − *F_c_* electron density map of the 5′-ppp dsRNA is shown in *blue* contoured at 2.5 σ, and the 2*F_o_* − *F_c_* electron density map of the active site residues are shown in *orange* contoured at 1 σ. *A,* only water molecules, but not Mn^2+^, are present in the active site prior to the soaking. *B,* after soaking in Mn^2+^ solution for 1 min, one Mn^2+^ was identified and was coordinated by three water molecules, side chain (OD1) of Asp-389 and two phosphoryl oxygen molecules (*O1P* and O3*) of the dsRNA substrate (supplemental Fig. S4). The electron density for the 5′-ppp moiety (*triphosphate*) of the cleaving could be detected. *C*, after soaking for 1.5 min, only one Mn^2+^ ion can be seen with three coordinating water molecules. *D,* after soaking in Mn^2+^ solution for 5 min, the electron density of the 1st Mn^2+^ appeared, and the electron density of the side chain of Tyr-429 disappeared, whereas other local residues did not change, suggesting that Tyr-429 became structurally disordered.

Although DEDDH nucleases are known to cleave ssRNA or DNA via the two-metal mechanism (25, 26), how LASV NP, a 3–5′ DEDDH exoribonuclease, degrades dsRNA is not clear. Structures of only a few known 3′-5′ exonucleases, such as Trex1, Trex2, and Klenow fragment, in complex with their DNA substrates are available (20, 22, 23), each using a catalytically inactive protein. The NP-C·dsRNA complex structure in this study contains an enzymatically active exoribonuclease domain of LASV NP and a dsRNA substrate in the catalytic pocket. This provides a unique opportunity to investigate the kinetic process of how this particular 3′-5′ exoribonuclease activity degrades dsRNA substrate. To do this, we set up a time course experiment to capture the conformational changes of the NP-C·dsRNA complex during the enzymatic reaction. The LASV NP-C·dsRNA complex was soaked in a cryoprotectant solution with 100 mm MnCl_2_, and the crystals were incubated for 1, 1.5, 5, or 10 min at room temperature under a standard crystallization condition but with pH 5.5 that would slow down the catalytic reaction for the purpose of capturing the different stages of the reaction (Fig. 6). Crystals that were soaked for 10 min were not good for data collection. Over the time course, the crystal cell volumes changed gradually from 474,825 Å^3^ to 444,643 Å^3^ (Table 1). Several changes were noted. First of all, the electron density signal of the 1st Mn^2+^ ion, which was visualized in the RNA-free Mn^2+^-bound NP structure (PDB code 3MWT), was not clear at first in the co-crystal structure but became visible over time. In contrast, the 2nd Mn^2+^ ion, which was not visible in the RNA-free NP structure (PDB code 3MWT), was now clearly visible and was coordinated by the side chain (OD1) of Asp-389, two scissile phosphoryl oxygen molecules (O1P and O3*) of the dsRNA substrate, and three water molecules (Fig. 6 and supplemental S4 and S5). Second, the electron density of the 5′-ppp moiety of the triphosphate dsRNA substrate became clearly visible, and that of an additional nucleotide C4 at the 5′ end of the complementary strand also became partially visible. This strongly suggests that the RNA molecule identified in the NP-C·dsRNA complex structure (Fig. 3) is indeed an intermediate product of the NP-C exoribonuclease activity. In addition, the electron density for the 4-bp dsRNA in the catalytic cavity became less clear over time, which serves as evidence for the dsRNA moving toward the catalytically active site during the cleavage reaction (Fig. 6*C* and supplemental Fig. S3). Last, the side chain of residue Tyr-429 became structurally disordered, whereas those of other local residues remained unchanged (Fig. 6*D*), indicating that the Tyr-429 residue moved from the G5 base to stack against the next C6 base of the complementary strand in the 5′ to 3′ direction (3′-CCCG*XXXX-*5′) when the cytidine monophosphate was removed from the 3′ end of the cleaving strand (5′-GGGC-3′).

**TABLE 1:** **Data collection and structure refinement statistics**

Data collection	LASV NP-C·dsRNA	LASV NP-C·dsRNA Mn^2+^ 1 min	LASV NP-C·dsRNA Mn^2+^ 1.5 min	LASV NP-C·dsRNA Mn^2+^ 5 min	TCRV NP-C
Wavelength (Å)	0.87260	0.9763	0.9763	0.9763	0.9798
Resolution (Å)	39.22-1.99 (2.10-1.99)*^a^*	52.24-1.68 (1.77-1.68)	69.54-1.98 (2.08-1.98)	73.90-2.46 (2.60-2.46)	57.58-1.73 (1.82-1.73)
Space group	P4_1_2_1_2	P4_1_2_1_2	P4_1_2_1_2	P4_1_2_1_2	I4

**Unit cell**					
*a*, *b*, *c* (Å)	*a* = 46.88	*a* = 46.58	*a* = 46.51	*a* = 44.78	*a* = 115.17
α, β, γ (°)	*b* = 46.88	*b* = 46.58	*b* = 46.51	*b* = 44.78	*b* = 115.17
α = β = γ = 90°	*c* = 216.60	*c* = 208.82	*c* = 208.66	*c* = 221.74	*c* = 56.92
Unique reflections	17,730 (1218)	27,463 (3895)	17,097 (2421)	8,951 (1246)	39,191 (5674)
Redundancy	6.8 (6.5)	11.4 (12.2)	7.2 (7.4)	8.2 (8.1)	5.8 (5.4)
*I*/σ	18.0 (4.8)	15.9 (5.2)	18.4 (8.2)	14.2 (4.5)	10.5 (1.9)
Completeness (%)	100 (99.6)	100 (100)	99.9 (100)	99.9 (100)	99.9 (100)
Cell volumes (Å^3^)	474,825.68	453,075.59	451,347.47	444,643.56	754,994.188
Solvent content (%)	52.33	49.61	49.42	50.70	46.39
Wilson *B* factor	33.12	20.59	26.48	48.22	27.78
*R*_merge_*^b^*	0.111 (0.536)	0.084 (0.489)	0.062 (0.217)	0.078 (0.407)	0.084 (0.785)

**Refinement**					
Number of atoms					
Protein	1621	1636	1636	1564	1565
RNA	172	181	181	202	0
Mn^2+^/Zn^2+^	0/1	1/1	1/1	2/1	0/1
Solvent	100	194	154	12	137
Mean *B*-factors (Å^2^)					
Protein	30.81	26.50	36.10	24.24	23.17
RNA	36.90	27.30	46.02	25.20	0
Mn^2+^/Zn^2+^	0/15.05	26.08/14.3	38.30/25.7	25.25/28.0	0/24.81
Solvent	39.50	34.58	40.20	39.02	29.12
*R*_factor_	0.2032	0.1988	0.1877	0.2432	0.1532
*R*_free_	0.2404	0.2238	0.2289	0.2888	0.1668
Rmsd bonds (Å)/angels (°)	0.011/1.290	0.010/1.157	0.008/1.537	0.010/1.15	0.010/1.252
Residues in Ramachandran core (%)	98.00	97.00	97.00	97.00	96.00
PDB code	4G9Z	4GV3	4GV6	4GV9	4GVE

*^a^* Values in parentheses are the highest resolution shell.

*^b^ R*_merge_ = Σ_hkl_Σ|*I_i_* − (*I*)|/Σ*_hkl_*Σ*_i_I_i_*, where *I_i_* is an intensity for the *i*th measurement of a reflection with indices *hkl* and (*I*) is the weighted mean of the reflection intensity.

## DISCUSSION

Arenaviral NPs are the newest members of the 3′-5′ DEDDH exoribonuclease family and their RNase activities play an unprecedented role in inhibiting type I IFN production (8). We have shown that NP exoribonuclease preferentially degrades dsRNA templates *in vitro* (Fig. 1), which is consistent with the known role of triphosphate dsRNA as the pathogen-associated molecular pattern RNA that triggers the type I IFN production (27, 28). We therefore propose a novel immune evasion mechanism in which NP suppresses the IFN induction by selectively degrading these immune-stimulatory RNAs. In this study, we provide atomic views of the active form of this unique exoribonuclease enzyme of the LASV NP protein in the process of trimming the 3′ end of its preferred dsRNA substrate.

While our manuscript was in preparation, Hastie and colleagues (12) published the structure of a catalytically inactive version of LASV NP-C in complex with a dsRNA at 2.9 Å. At this resolution, it was not possible to visualize the sequence of the dsRNA and water molecules in the active site as well as to identify all possible side chain interactions. As such, only two residues (Gly-392 and Tyr-429) were identified to be essential for binding to and processing dsRNA substrate. Our studies with a catalytically active enzyme have revealed several mechanistic insights into this unique NP exoribonuclease activity. First of all, the NP catalytic cavity binds the 4-bp RNA duplex and directly positions the 3′-end of the cleaving strand in the catalytically active site (Fig. 3). The cleaving strand of the RNA substrate directly or indirectly interacts with mostly conserved residues (Gly-392, Glu-391, Asp-466, Arg-492, Gln-462, and Ser-430) of the NP protein, whereas the complementary strand interacts with the non-conserved residues (Tyr-429, Arg-393, Gln-425, Asp-426, Gln-422, and Asp-465) (Fig. 3), perhaps allowing for the flexibility of NP binding the different dsRNA substrates. Mutational analyses suggest that the conserved residues are indeed important for the NP exoribonuclease activity, whereas the non-conserved residues can tolerate alanine substitutions without affecting the enzymatic and biological functions (Fig. 4). Therefore, the strict requirement of several residues in positioning the cleaving strand and the flexibility in binding the complementary strand provide not only the structural conservation that is important for this unique dsRNA-selective enzymatic mechanism but also the necessary flexibility needed to process any forms of dsRNA substrates that can potentially trigger IFN production. It is important to note that in addition to the five catalytic residues, the Arg-492 residue is indispensable for exoribonuclease activity. We propose that Arg-492, in addition to positioning the 3′ ribonucleotide, may be directly involved in the cleavage step.

Furthermore, the 3′ end ribonucleotide in the NP-C·dsRNA complex structure remained in a double-stranded form, although directly presented to the catalytic residues for catalysis. In contrast, other known 3′-5′ exonucleases such as Trex1, Trex2, and Klenow fragment contained essentially single-stranded nucleic acid in their catalytic pockets, even though Trex1 showed a preference for the dsDNA substrate (29) and Klenow was co-crystalized with a hairpin (ds) DNA (23). We obtained a trapped intermediate product complex of LASV NP-C in the process of cleaving dsRNA that reveals a unique mechanism by which NP exoribonuclease activity cleaves the 3′ ribonucleotide prior to separating the double-stranded form. Most known 3′-5′ exoribonucleases rely on ATP-dependent helicase activity to unwind RNA duplexes to promote exonucleolytic activity, with the exception of *E. coli* RNase R (30).

**FIGURE 7.**
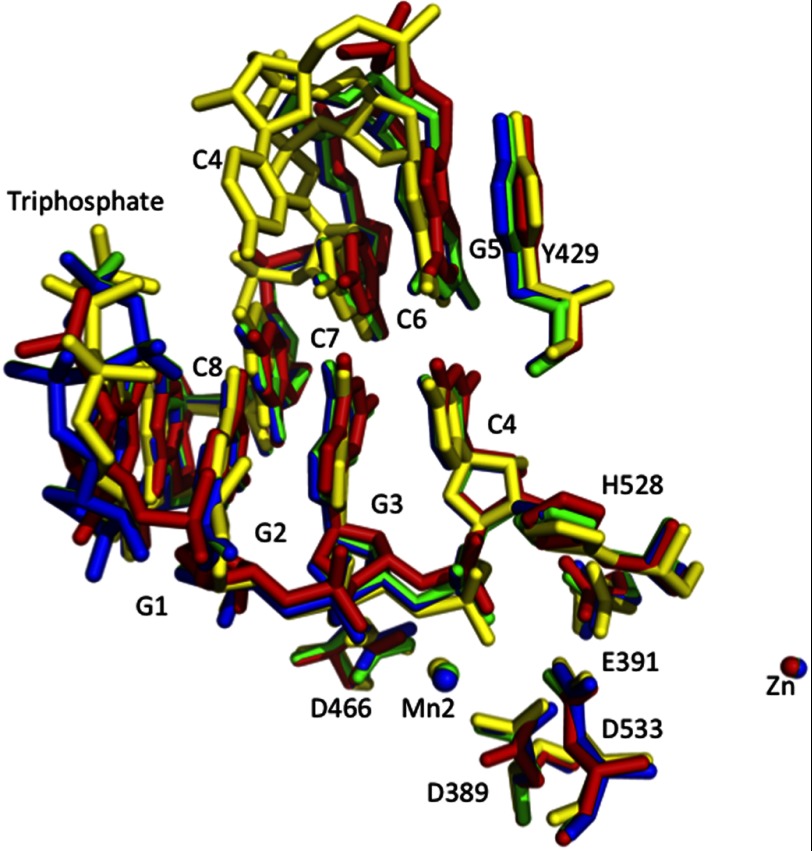
**Conformational changes observed for the 5′-ppp dsRNA and metal ions relative to the five catalytic residues over the time course of the soaking experiment.** These molecules before and 1, 1.5, and 5 min after soaking are shown in *red*, *green*, *blue*, and *yellow*, respectively. Over the time course, the 3′-end ribonucleotide C moved downward and rotated in a counterclockwise direction, which caused the scissile bond to bend for cleavage (supplemental Fig. S6). The G base of the complementary strand also rotated in a counterclockwise direction. Together, these movements may dissociate the base pair after the 3′ end ribonucleotide was cleaved. In addition, the Mn^2+^ ion changed position considerably over the soaking process, whereas the Zn^2+^ ion remained largely undisturbed.

We have also conducted a time course soaking experiment to explore the kinetic process of the LASV NP exoribonuclease function (Figs. 6 and 7 and supplemental Figs. S4 and S6). Taken these data together, we propose that the LASV NP exoribonuclease processes dsRNA substrate in a four-step mechanism (1). NP positions the 3′ end ribonucleotide into the active site, through the dsRNA-binding interface (2). NP then uses the 5 catalytic residues (Asp-389, Glu-391, Asp-466, Asp-533, and His-528) and Arg-492 to break the phosphodiester bond of the 3′ ribonucleotide from the cleaving strand (in its double-stranded form), via a well known two-metal mechanism (3). The 3′ end ribonucleotide is then dissociated from the base pair formation with the complementary strand, possibly through interactions with residues Tyr-429, Ser-430, Asp-426, and Arg-393 (4). The cleaved nucleotide monophosphate is released from the active site.

Our current study also challenges a long-standing belief that TCRV NP is unique among the arenaviral NPs in its lack of IFN suppressive function. A previous study by Martínez-Sobrido *et al.* (7) has shown that the NP proteins from various arenaviruses of both Old World and New World groups, including LASV, lymphocytic choriomeningitis virus (LCMV), Whitewater Arroyo virus (WWAV), Pichinde virus (PICV), Junin virus (JUNV), Machupo virus (MACV), and Latino virus (LATV), can effectively inhibit IFN production, with a notable exception of TCRV, the only known arenavirus isolated from bats (1). Nevertheless, we have unequivocally shown here that TCRV NP resembles LASV NP in tertiary structure, exoribonuclease activity *in vitro*, as well as in its ability to suppress Sendai virus-induced IFN production in cell culture (Fig. 5). We have also produced preliminary data to show that cells infected with Tacaribe virus produce similarly low levels of type I IFNs as seen with those infected with LCMV and PICV (data not shown). The NP gene used in the current study was provided by J. Nunberg, University of Montana, and was also amplified from Tacaribe virus purchased from ATCC (strain 11573). It is not clear what source of TCRV NP gene was used in the original report, however (7). Therefore, the discrepancy between the findings by Martínez-Sobrido (7) and ours needs to be investigated further. Regardless, we have provided in the current study compelling structural, enzymatic, and biological evidence to demonstrate that TCRV NP has 3′-5′ exoribonuclease activity and IFN suppressive ability as those of the LASV NP. We therefore believe that the NP RNase activity-mediated inhibition of the IFN production via degradation of the immune-stimulatory RNAs is a common mechanism among all known arenaviruses.

In summary, we have provided the structural basis for a distinctive mechanism of a new member of 3′-5′ DEDDH exoribonuclease family, LASV NP, in binding and cleaving a dsRNA substrate, and have shown that suppression of IFN production via NP RNase activity by degrading immune-stimulatory RNAs represents a common mechanism of arenaviruses to mediate innate immune evasion. These new discoveries may help stimulate the development of specific therapeutics against these important viral pathogens that can cause high mortality and morbidity in humans.

## Footnotes

6 J. Nunberg, personal communication.

## Footnotes

5 The abbreviations used are:

LASV: Lassa fever virus
TCRV: Tacaribe virus
NP: nucleoprotein
nt: nucleotide
PDB: Protein Data Bank.

## Addendum

As this manuscript was in review, an independent study has been published by Harmon *et al.* (31) to describe the role of TCRV NP in suppressing IFN-β production, which reinforces our finding.
